# Hyaluronic acid treatment versus standard of care in chronic wounds in a German setting: Cost‐effectiveness analysis

**DOI:** 10.1002/hsr2.969

**Published:** 2022-12-02

**Authors:** Dominik Blunck, Oliver Schöffski

**Affiliations:** ^1^ Department of Health Management, Institute of Management Friedrich‐Alexander‐Universität Erlangen‐Nürnberg (FAU) Nuremberg Germany

**Keywords:** chronic wounds, cost‐effectiveness analysis, dressings, hyaluronic acid, silver

## Abstract

**Background and Aims:**

Chronic wounds are a major burden for worldwide health care systems. In the management of chronic wounds several strategies with innovative and active agents emerged in the past few years, such as hyaluronic acid containing wound dressings. Evidence comparing the cost‐effectiveness of hyaluronan and standard of care dressings (hydrofiber with silver) is still missing. The aim of the study is thus, to assess the cost‐effectiveness of hyaluronan versus standard of care dressings (hydrofiber with silver) in chronic wounds from a German statutory health insurance perspective.

**Methods:**

A decision tree was modeled to quantify the cost and healing rate at 12 weeks for the hyaluronan and silver dressings strategies. Input parameters were collected literature‐based, accounting for healing rates, dressing prices and prices for dressing changes and associated home care. Parameter uncertainty was accounted for by one‐way and probabilistic sensitivity analysis.

**Results:**

Hyaluronic acid showed a better healing rate (60.68%) and noticeable lower cost (749.80 Euro) compared to standard of care (silver containing) dressings (59.62%; 883.05 Euro), resulting in an Incremental Cost Effectiveness Ratio of −12,570.57. The hyaluronan approach is hence a dominant strategy in chronic wound management. Sensitivity analysis confirmed these results, giving a range of 60%– 70% of cost‐effective scenarios.

**Conclusions:**

Hyaluronic acid dressings showed to be a clinical more effective strategy at significantly lower cost in chronic wounds compared to standard of care (hydrofiber with silver).

## 1 INTRODUCTION

### 1.1 Chronic wounds

The relevance of chronic wounds is increasing, especially because chronic wounds are associated with a high cost‐of‐illness.^1^, ^2^ Chronic wounds are often facilitated by specific underlying diseases, such as Diabetes mellitus, pressure ulcers or infections.^3^, ^4^ Diabetes mellitus associated circulatory disorders, for example, make even small wounds hard to heal and hence chronical.^5^ Demographic change and an increasing incidence of Diabetes mellitus points out the rising relevance of chronic wounds.^6^ The definition of chronic wounds, however, is not consistent in literature. One approach defines wounds as chronic if they do not heal within 8 weeks of therapy.^7^, ^8^ Other approaches define wounds already as chronic if they do not heal within 3 weeks or if they are described as hard to heal.^9^

Depending on the stage of wound healing and the complexity of its presentation, different therapies can be followed. Besides debridement, antimicrobial substances or negative pressure, different types of wound dressings are used. For example, there exist moistened gauze, foam dressings, algal‐fibers, activated carbon, or several dressings with active agents.^6^, ^7^ In the German setting chronic wounds are often treated by ambulatory care services. Ambulatory care nurses visit patients, for example, on a daily basis and change dressings if required.

One ingredient of active wound dressings that is applied onto the wound dressing is hyaluronic acid (HA). HA is a polymer occurring in different tissues of the human body naturally. It contributes to wound healing by providing supportive structures for the extracellular matrix which acts as a base structure for the healing tissue.^10^ Furthermore, HA improves wound healing by increasing the level of specific growth factors in the wound milieu,^11^ which decreases time to heal and reduces scars.^12^, ^13^ A systematic review and meta‐analysis from 2012 finds that various end points such as wound healing, time to heal, wound area or granulation are improved in eight of 10 studies by the application of HA.^14^ Another meta‐analysis from 2016, however, argues that the pooled evidence of overall superiority of HA is still limited, as they did not find clearly higher levels of complete wound healing rates, although pain intensity was clearly lower in HA.^15^ Since 2016 further research was conducted, showing accelerated wound healing in the HA group (see Table 1).

**Table 1:** Effectiveness of HA dressings

Study	Design	Primary endpoint	Secondary endpoint	Dressing	*n*	Results
Alvarez et al. (2017)^16^	Parallel group, randomized controlled trial	Complete healing W12, W16	Time to heal	IG: HA + multilayer compression	9	Healing W12: 67%, W16: 88%, time to heal: 41 days
				CG: Nonadherent dressing + multilayer compression	7	Healing W12: 14%, W16: 43%, time to heal: 104 days
DeCaridi et al. (2016)^17^	Parallel group, open label trial	Complete healing W6	Wound size reduction 6 weeks	G1: Polynucleotides and HA	12	Healing: 60%, reduction: 67%
				G2: Only HA	4	Healing: 22%, reduction: 34%
Gazzabin et al. (2019)^18^	Monocentric, prospective, open label trial	Wound size reduction D1, D7, D28			25	D1: 3%, D7: 34%, D28: 85%
Mikosinski et al. (2021)^19^	Multicenter, prospective, double‐blind randomized controlled trial	Complete healing W23	Residual area compared to baseline, analgetics utilization, pain intensity, adverse events	IG: HA	83	Healing: 40%, residual area: 41%, analgetics utilization: 45%/62 doses, pain reduction W0 vs W23: 21 VAS points, adverse events: 34%
				CG: Gauze	81	Healing: 19%, residual area: 82%, analgetics utilization: 42%/61 doses, pain reduction W0 vs W23: 13 VAS points, adverse events: 41%
Scalise et al. (2017)^20^	Prospective, double‐blind, randomized controlled trial	Mean debridement rate D15	Pain reduc., wound size reduc., periwound skin status	IG: HA Collagenase	58	Full debridement D7: 19%, D15: 47%, D21: 53%, D30: 63%
				CG: Placebo	55	Full debridement D7: 5%, D15: 10%, D21: 26%, D30: 40%
Segreto et al. (2020)^21^	Prospective, single‐blind, randomized controlled trial	Wound size reduction D30		G1: Polyurethan with silver	10	2%
				G2: Porcine dermis	10	21%
				G3: Polynucleotides and HA	10	74%
Stryja et al. (2022)^22^	Multicenter, prospective, open label, randomized controlled trial	Change in wound bed slough W3	Wound size reduction, changes in wound bed tissue composition, investigator satisfaction, exudation level, surrounding tissue composition, pain	IG: HA + Octenidine	48	Change slough W3: 40%, wound size reduction: 11 cm^2^, wound bed tissue composition: no sign. diff., improved infections: 56%, investigator satisfaction: 75%, exudation: no sign. diff., surrounding tissue: sign. improved, pain: no diff.
				CG: Silver‐containing dressings	39	Change slough W3: 49%, wound size reduction: 5 cm^2^, wound bed tissue composition: no sign. diff., improved infections: 72%, investigator satisfaction: 66%, exudation: no sign. diff., surrounding tissue: not sign. improved, pain: no diff.

Abbreviations: CG, control group; D1–30, Day 1–30; Diff., Difference; G1–3, Group 1–3; HA, hyaluronic acid; IG, intervention group; W1–23, Week 1–23; Sign, statistically significant.

HA showed a positive effect to the wound environment and quicker tissue regeneration in different studies.^23^, ^24^ For example, there are different randomized controlled trials (RCT) that show clinical effectiveness of HA containing wound dressings, compared to non‐active wound dressings.^14^, ^16^, ^21^, ^25^ Compared to standard gauze dressings, HA shows lower pain intensity during dressing changes.^25^ Additionally, to HA's properties to promote wound healing, combination with an antiseptic agent—for example, Octenidine—provides the ability to fight bacteria to these dressings, which is important in chronic and infected wounds.^26^, ^27^ In addition to quicker wound healing, HA dressings also come with lower change‐frequencies,^28^ which is not only economically preferable due to lower numbers of units utilized, but also might again reduce patients' pain from dressing changes.

Another ingredient for active dressings is silver. Although there is evidence that silver has cytotoxic properties that might hinder healing, silver is a standard therapy for chronic wounds,^26^, ^29^, ^30^ especially in infected wounds due to its antimicrobial effects.^31^ Similar to HA dressings, also silver dressings showed effectiveness compared to non‐active dressings.^32^ Silver dressings are suitable for infected wounds as silver has antibacterial properties.^29^ In case of low change frequency, however, there is a risk of silver being incorporated by the surrounding tissue.^26^

In the management of chronic wounds, both HA and silver dressings show better outcomes in effectiveness than dressings not containing active ingredients.^33^ In recent years, there have been calls to extend the view of wound care from an only‐effectiveness perspective towards an efficiency perspective.^34^ Several studies touch health economic stand points by reporting, for example, dressing wear time,^32^, ^35^ ignoring that large price differences can undermine the effect of shorter wear time. Another study already provide evidence that silver dressings are cost‐effective compared to non‐active standard dressings by conducting a dedicated economic evaluation.^22^, ^36^ Although large‐scale, direct comparing meta‐evidence is still missing, there is evidence suggesting that HA shows better clinical effectiveness compared to silver in chronic wounds. A recent RCT compared the effectiveness of HA with silver dressings and did find better wound size reduction and erythema reduction in the HA arm, as well as no superiority of silver in any other endpoint.^22^ A cost‐effectiveness analysis comparing HA and silver dressings, however, is still missing.

### 1.2 Aim

In the past, HA wound dressings—compared to other wound dressings containing active ingredients—were very cost‐intensive and put a strain on the budgets of German physicians. Modernized manufacturing processes allow hyaluronic acid products to be produced at prices that are generally lower than those for silver‐containing wound dressings. There is all the more interest in evaluating the cost‐effectiveness of these two wound therapy strategies. Therefore, the aim of this study is, to close the aforementioned research gap by comparing and quantifying the cost‐effectiveness of HA containing wound dressings versus silver containing wound dressings in chronic wounds. Thereby, this study provides evidence to allow efficient decisions by decision makers and caregivers.

## 2 METHODS

To answer the research question, we conducted a cost‐effectiveness analysis from a German statutory health insurance (SHI) perspective. The economic evaluation was conducted according to the Consolidated Health Economic Evaluation Reporting Standards 2022 (see the Supporting Material for the reporting checklist).^37^ In the following, the decision tree model, input parameters, and analyses are explained.

### 2.1 Decision tree

**Figure 1:**
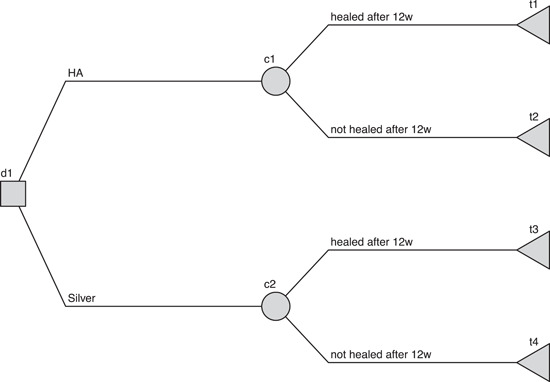
Decision tree

A decision tree is a suitable and accepted method of assessing cost‐effectiveness in the given setting^36^ as it is reflects the relatively simple decision scenario in a short‐term time frame.^38^ The decision tree is displayed in Figure 1. The square *d1* is the decision between HA dressing and silver dressing. Circles *c1* and *c2* are chances for healing in the silver dressing arm and HA arm, respectively; Triangles *t1* to *t4* are terminal nodes representing outcomes. Contrary to Jemec et al.,^36^ we only incorporate two health states (healed and not healed), instead of healed, healing, and not healed. We did not find enough evidence in literature research to replicate the approach of Jemec et al.^36^ Furthermore, differentiation between healing and healed/not healed might be sensitive to errors in clinical trials.

### 2.2 Data

Input data for the cost‐effectiveness analysis (CEA) model were obtained by literature search in Medline/Pubmed, Cochrane Library, Science‐Direct, and Scopus using the search terms hyaluronic acid AND dressing AND chronic wound, respectively silver AND dressing AND chronic wound, as well as the associated MeSH‐terms where applicable. Studies that address chronic wounds and provide data within the time horizon of our primary effectiveness endpoint (see below) were included. We hereby chose publications with the highest evidence level (i.e., meta‐analysis before randomized‐controlled trial). An overview of input data is provided in Table 2.

**Table 2:** Input data

Parameter	Baseline value	OWSA	PSA	Source
Price HA (per unit)	19.0750	+/− 5%	0.0477; 400^a^	Sellmer (2021)^39^
Price Silver (per unit)	24.6270	+/− 5%	0.0616; 400^a^	Sellmer (2021)^39^
Units per week HA	2	2–3	8; 0.18^b^	Lee et al. (2016)^40^
Units per week Silver	2	2–5	8; 0.23^b^	Dimakakos et al. (2009),^41^ Vanscheidt et al. (2003),^42^ Jemec et al. (2014)^36^
12w healing rate HA	0.6068	0.24–0.96	2.03; 1.2960^c^	Chen et al. (2014)^43^
12w healing rate Silver	0.5962	0.07–0.64	1.82; 1.4323^c^	Rodriguez‐Arguello et al. (2018)^44^
Price dressing change	12.1667	9.14–14.44	0.5264; 213.55^a^	Tariffs according to framework agreement^45^, ^46^, ^47^

*Note*: Prices in 2021 Euro.

Abbreviations: OWSA, one‐way sensitivity analysis; PSA, probabilistic sensitivity analysis.

^a^ Parameters theta and kappa of gamma distribution.

^b^ Parameters *n* and *p* of binomial distribution.

^c^ Parameters alpha and beta of beta distribution.

#### 2.2.1 Costs

To assess the cost of therapy for 12 weeks, the prices for dressings, the number of dressings per week and the prices for dressing changes were collected. After calculating the cost per week, it was multiplied by 12. All prices are reported as 2021 Euros.

Prices for wound dressings were retrieved from a price list of Sellmer (2021),^39^ which is released annually and contains dressing prices from the German SHI perspective. Prices for HA dressings (Sorelex HA) are 19.075 Euro per unit and 24.627 Euro per unit for silver dressings (Aquacel Ag + Extra).

The number of dressings used per week is hardly described in literature. As most studies evaluate the effect of a certain dressing, utilization of dressings is often prescribed by the study protocol. Real‐world‐data could not be found in literature search. For the HA arm, however, we identified studies that changed dressings 2–3 times per week.^40^ For the silver dressing arm, we found studies that changed dressings 2–3 times per week, too.^41^, ^42^, ^48^ A cost‐effectiveness analysis reported silver dressings changes for chronic wounds of 2–4 times per week (2–5 times in sensitivity analyses).^36^

Prices for dressing changes, which include wound care, in Germany are negotiated between the National Association of Statutory Health Insurance Funds and the head organizations of home care nursing services as a framework agreement.^49^ Therefore, the mean value of prices from public available framework agreements was used for this analysis.^45^, ^46^, ^47^, ^50^

#### 2.2.2 Effectiveness

Literature research did not give suitable studies that report quality‐adjusted life years (QALYs) in both treatments. QALYs are required to conduct a cost‐utility analysis which allows comparison of health‐technologies across indications.^51^ As QALYs are not available, we focused on a unidimensional and relevant endpoint to assess cost‐effectiveness of HA versus silver‐containing dressings. We found complete healing after 12 weeks as a common endpoint in studies assessing the effectiveness of wound healing.^14^, ^43^, ^52^ Different to other end points, such as wound are reduction or pain, complete wound healing is considered to be “more established, objective, and quantifiable.”^15^ ^,p.590^ Therefore, we operationalized effectiveness as healing probability after 12 weeks of treatment.

Due to missing comparative studies, healing rates for HA and silver were identified by separate meta‐analyses assessing the use of the specific therapy in chronic ulcers. Healing rates for HA are calculated as the number of patients with complete healing at 12 weeks *n* = 125 divided by the total number of patients treated with HA *n* = 206. This procedure yielded a complete healing rate of 60.68% after 12 weeks.^43^ For silver dressings, data was retrieved from a scoping review and a meta‐analysis^44^, ^53^ which follow the PRISMA guidelines. The number of patients with complete healing *n* = 148 was divided by the number of patients treated with silver dressings *n* = 254. This way, a complete healing rate of 58.27% after 12 weeks was calculated for silver dressings. The acceleration of wound healing in HA, which has been stated in some studies, was conservatively disregarded and is discussed in the limitations section.

### 2.3 Analysis

To control for parameter uncertainty, deterministic and probabilistic sensitivity analysis (PSA) were conducted. In case of deterministic sensitivity analysis, a one‐way sensitivity analysis (OWSA) was performed by rerunning the model changing each parameter to a minimum and maximum while keeping all other parameters fixed (see minimum and maximum parameters in Table 2, column OWSA). Parameter ranges for unit prices were assumed to be 5% above/below the best‐case scenario. Ranges for dressing utilization were identified in literature.^36^, ^40^, ^41^, ^42^ The healing probabilities are derived from meta‐analyses used for the bet‐case scenario. Ranges are the minimum and maximum of healing rates found in each meta‐analysis.^43^, ^44^ Ranges for dressing change‐prices are minimum and maximum values identified in the regional framework agreements.^45^, ^46^, ^47^

In case of PSA, a Monte‐Carlo‐simulation with 10,000 replications^54^ was performed that drew parameter values from the distributions reported in Table 2. Prices (i.e., dressing prices and prices for dressing changes) were assumed to be gamma distributed because values below 0 are impossible. Number of units utilized is a positive number as well. Furthermore, it is a discrete scale of natural numbers as only full units can be billed. Hence, the distribution of number of units utilized was assumed to be binomial. Healing probabilities are beta distributed as probabilities are defined between 0 and 1. Based on this, the cost‐effectiveness acceptability curve was built which displays the share of cost‐effective scenarios depending on the willingness to pay.

All analyses were conducted in R statistics software,^55^, ^56^ using packages dplyr^57^ and tidyr^58^ for data management, rdecision^59^ for visualization of the decision tree (Figure 1), and ggplot2^60^ for all other plots. All data used and analyzed is presented and described in the paper.

### 2.4 Ethical approval and public involvement

This study does not involve human subjects or include any primary data and only uses secondary data from already published studies. Therefore, ethical approval was waived. Furthermore, patients, the general public, clinicians, or payers were not engaged in the design of the study.^37^

## 3 RESULTS

In following, first the results of the best case point estimates are presented before robustness of the results against parameter uncertainty is tested.

### 3.1 Best case analysis

Table 3 reports the results of the economic evaluation of HA versus silver containing dressings in chronic wounds for complete healing after 12 weeks as our best estimate. The cost in the HA arm is 749.80 Euro and thus lower than the cost in the silver arm (883.05 Euro). Furthermore, effectiveness, that is, complete healing rate after 12 weeks, is nearly identical, yet higher in HA than silver patients (60.68% vs. 59.62%). Accordingly, the Incremental Cost Effectiveness Ratio (ICER) can be computed by dividing the—in this case negative—incremental cost by the—in this case positive—incremental effectiveness, resulting in an ICER = −12,570.57 (see Table 3). The ICER indicates the cost associated with an incremental unit of effectiveness. The Interpretation of a negative ICER, however, is not easily possible, anyhow it is in favor of the intervention.

**Table 3:** Results

HA		Silver				
Cost	Complete healing rate	Cost	Complete healing rate	Incremental cost	Incremental healing	ICER
749.80	0.6068	883.05	0.5962	−133.25	0.0106	−12 570.57

*Note*: Costs in 2021 Euros.

### 3.2 Sensitivity analysis

**Figure 2:**
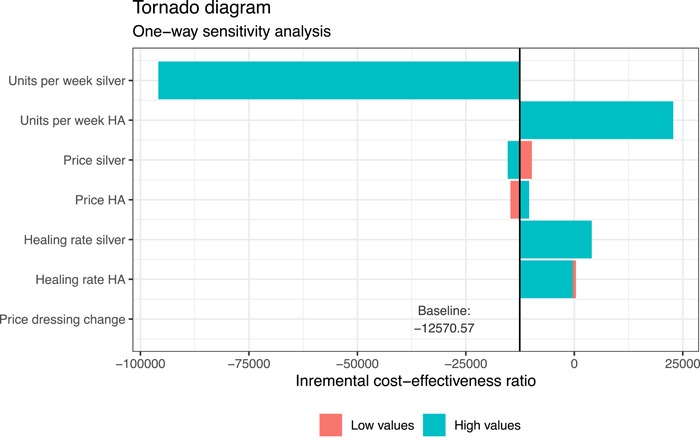
One‐way sensitivity analysis

Results of OWSA are displayed in a tornado diagram in Figure 2. The OWSA shows that results are majorly driven by the number of dressings utilized.

**Figure 3:**
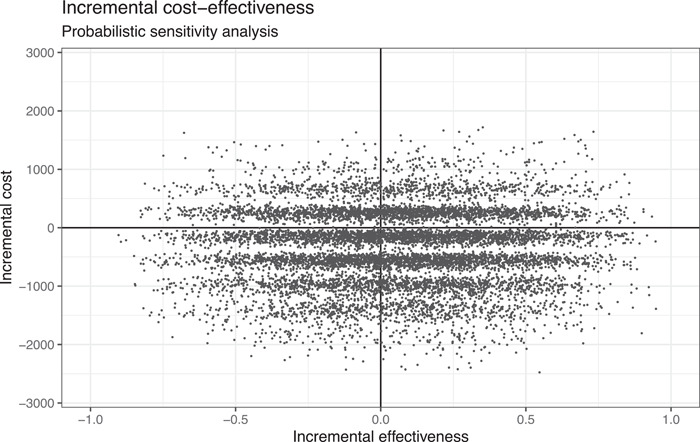
Probabilistic sensitivity analysis

Figure 3 provides the incremental cost‐effectiveness diagram of the sensitivity analysis after 10,000 replications, where each point represents the incremental cost‐effectiveness ratio of one iteration in the sensitivity analysis. For a given *y*‐value, a higher *x*‐value represents a better cost‐effectiveness—better outcome, same cost. Instead, a higher y‐value for a given x‐value indicates worse cost‐effectiveness—higher cost, same outcome. Values on the cost scale are clustered and appear as stripes because of the discrete distribution of numbers utilized. Hence, health technologies in the lower right quadrant would be regarded as cost‐effective—higher effectiveness, lower cost—and health technologies in the upper left would be regarded as not cost‐effective—lower effectiveness, higher cost. The upper right and lower left quadrants, however, represent scenarios which are a real trade‐off situation. In this case, usually the willingness to pay for a certain unit of incremental effectiveness determines if decision makers would regard a specific scenario as cost‐effective or not.

**Figure 4:**
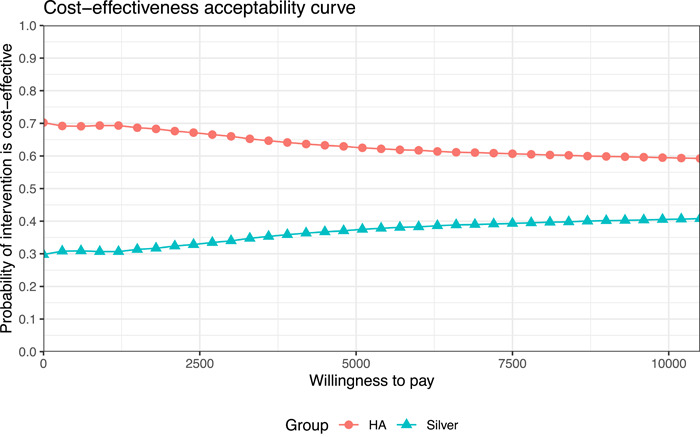
Cost‐effectiveness acceptability curve

Figure 4 shows the cost‐effectiveness acceptability curve which provides the probability that HA/Silver is cost‐effectiveness depending on the willingness to pay based on the Monte‐Carlo‐simulation. The HA curve (red dots) lies above the silver curve (blue triangles) at all willingness to pay‐levels. Starting at 70% cost‐effective probability of cost‐effectiveness for a willingness to pay of 0 Euro, it levels off at approximately 60% for a willingness to pay up to 10,000 Euro. Therefore, we imply that HA is a dominant strategy compared to silver, meaning that it is advantageous in terms of costs and provides better clinical outcomes. Sensitivity analysis supported our findings of the best case analysis. Furthermore, it should be noted that advantages in the cost‐effectiveness of HA is mainly driven by prices and slightly better effectiveness, compared to silver.

## 4 DISCUSSION

### 4.1 Principal findings

To answer the question of cost‐effectiveness of HA dressings versus silver containing dressings in chronic wound care, we conducted a literature‐based cost‐effectiveness analysis. We found that the strategy of HA is superior to silver dressings in chronic wounds. Often, health technologies that provide better effectiveness, come at higher cost. Then, health economic evaluations can provide guidance for policymakers and health care providers as willingness to pay is a critical parameter to decide whether a health technology can be regarded as cost‐effective or not. In the case of this study, a higher effectiveness comes with lower cost, which allows cost savings and improved care for patients with chronic wounds.

Previous studies found silver dressings to be cost‐effective compared to standard dressings.^36^ Under the light of our results, we imply that HA dressings might also be cost‐effective compared to standard dressings. Besides this indirect comparison, however, further direct evidence is needed. Additionally, further research is needed to assess cost‐utility of different wound therapies. As mentioned before, wound healing is a good end point for assessing the effectiveness of therapies for chronic wounds. However, there might be further end points worth to consider, such as pain or health related quality of life. Therefore, future studies incorporating QALYs would allow a generic comparison.

Our study chose complete healing as the primary endpoint on which the cost‐effectiveness analysis was based on. However, it is known that not all chronic wounds reach complete healing. This group of patients might view high levels of wound size reduction as a very positive result. Quality of life, thus, might be positively affected in this subgroup without reaching complete healing. Therefore, future clinical studies and health economic evaluations should incorporate subgroup analysis and further endpoints, for example QALYs, as mentioned afore.

### 4.2 Limitations

The present study comes with several limitations. As already mentioned, the lack of a direct comparison study, along with missing real world to validate the literature‐based parameter inputs, might limit the validity of the study. Especially missing evidence regarding the use of further resources, further care or cost from adverse events associated with the considered treatments is limiting the results of this study and is probably underestimating the real cost of both treatment arms. However, this is the first study to assess the cost‐effectiveness of HA versus silver containing dressings and addresses this research gap. Future research is needed to further analyze the cost‐effectiveness of chronic wound therapies. Furthermore, the study considers only the treatment of the chronic wounds, which are mostly not an isolated issue but come with complex underlying diseases. Costs that are associated with the management of the underlying disease were not considered in this study which underestimates the true treatment costs for patients with chronic wounds.

As the design is literature‐based our results might be biased by publication bias.^61^ However, as the aim of our study is to compare HA versus silver containing dressings, we do not suspect that publication bias is more prevalent in one therapy, so the overall interpretation does not change. However, we acknowledge that the presence of publication bias or differences in its expression could affect the results. Furthermore, literature‐based inputs—despite their high level of evidence—might be prone to trial effect, as some results published in clinical trials or meta‐analyses might be biased by trial protocols or further trial effects, for example, higher adherence due to trial participation.^62^ Therefore, further evidence from a real‐world setting is needed.

Another limitation is the underlying assumption of equal time‐dependent distribution of cost in both interventions. As the primary endpoint chosen in this CEA is healed after 12 weeks and cost is viewed from this point in time, we implicitly assume that cost is equally distributed. However, there might be differences in overall treatment cost if in one therapy a large proportion of healed patients would be already healed after 1 week – and thus not generate further cost—and patients in the other therapy arm for example would be healed after 11 weeks—and thus generate more costs. The model ignores this fact and assumes that the distribution of healed patients over time is equally. This shortcoming is a common problem in CEAs and prevalent in comparable studies.^36^ However, we did not find enough evidence in literature to cope with this problem within the CEA model. We did find evidence, though, that suggests that healing might be quicker in HA, which might be due to cytotoxic effects of silver.^26^ Thus we regard our results as overestimating the cost of HA and underestimating the cost‐effectiveness by tendency. To address this limitation, more evidence is needed, for example in form of directly comparing randomized controlled trials or real‐world‐evidence.

Furthermore, the study might miss aspects about the healthcare context or that are relevant to stakeholders to apply the results in their specific role as we did not engaged patients, general public, clinicians, or payers in the design of the study.^37^

### 4.3 Conclusions

This study is the first to describe the cost‐effectiveness of hyaluronic acid dressings versus standard of care (hydrofiber with silver) dressings. The literature‐based decision tree analysis indicated that hyaluronic acid dressings come with slightly higher effectiveness and at lower costs. Care givers should consider health economic evidence in daily care to allow efficient resource allocations and improved outcomes for healthcare systems.

## AUTHOR CONTRIBUTIONS

**Dominik Blunck**: Conceptualization; Formal analysis; investigation; methodology; project administration; validation; visualization; writing – original draft; writing – review and editing. **Oliver Schöffski**: Conceptualization; Supervision; validation; writing – review and editing.

## CONFLICT OF INTEREST

The authors received funding for this study.

## TRANSPARENCY STATEMENT

The lead author Dominik Blunck affirms that this manuscript is an honest, accurate, and transparent account of the study being reported; that no important aspects of the study have been omitted; and that any discrepancies from the study as planned (and, if relevant, registered) have been explained.

## Supporting information

Supporting information.

## Data Availability

Data sharing not applicable—no new data generated.
